# A Systematic Review and Quality Evaluation of Studies on Long-Term Sequelae of COVID-19

**DOI:** 10.3390/healthcare10122364

**Published:** 2022-11-24

**Authors:** Jorge Vásconez-González, Juan S. Izquierdo-Condoy, Raul Fernandez-Naranjo, Esteban Ortiz-Prado

**Affiliations:** 1 One Health Research Group, Faculty of Health Science, Universidad de Las Américas, Quito 170507, Ecuador; 2 Health Management and Research Area, Universidad Internacional Iberoamericana, Arecibo, PR 00613, USA; 3 Facultad de Ciencias de la Salud, Universidad Latina de Costa Rica, San José 11501, Costa Rica

**Keywords:** sequelae, COVID-19, SARS-CoV-2, long COVID-19, systematic review

## Abstract

COVID-19 made its debut as a pandemic in 2020; since then, more than 607 million cases and at least 6.5 million deaths have been reported worldwide. While the burden of disease has been described, the long-term effects or chronic sequelae are still being clarified. The aim of this study was to present an overview of the information available on the sequelae of COVID-19 in people who have suffered from the infection. A systematic review was carried out in which cohort studies, case series, and clinical case reports were included, and the PubMed, Scielo, SCOPUS, and Web of Science databases were extracted. Information was published from 2020 to 1 June 2022, and we included 26 manuscripts: 9 for pulmonary, 6 for cardiac, 2 for renal, 8 for neurological and psychiatric, and 6 for cutaneous sequelae. Studies showed that the most common sequelae were those linked to the lungs, followed by skin, cutaneous, and psychiatric alterations. Women reported a higher incidence of the sequelae, as well as those with comorbidities and more severe COVID-19 history. The COVID-19 pandemic has not only caused death and disease since its appearance, but it has also sickened millions of people around the globe who potentially suffer from serious illnesses that will continue to add to the list of health problems, and further burden healthcare systems around the world.

## 1. Introduction

More than two years have passed since the emergence of a new zoonotic coronavirus [1]. Coronaviruses are infectious particles that are characterized by having a genome of the positive sense single-stranded RNA type: these are one of the largest groups of viruses that exist [2,3]. These viruses belong to the Nidovirales order, specifically in the Coronaviridae family, which is divided into two subfamilies, Letovirinae and Orthocoronavirinae, which contain 1 and 4 genera, respectively [3]. Currently, three coronaviruses have been detected that passed from animals to humans, that is, they are zoonotic diseases. The first was severe acute respiratory syndrome (SARS), first detected 20 years ago in China in 2002; ten years later the respiratory syndrome emerged from the Middle East (MERS) detected in 2012 in Arabia; and finally the SARS-CoV-2 was detected at the end of 2019 [4,5,6].

This disease emerged in China after close contact with mammals from a wet market in Wuhan [7]. The rapid spread of the SARS-CoV-2 virus took the planet by surprise, causing a severe flu-like illness that affected more than 670 million and killed more than 6.7 million worldwide. Of the individuals affected, about 80% had a mild to moderate disease, and among those with a severe disease, 5% developed critical illness [8]. The impact of SARS-CoV-2 should not be attributed to severe pneumonia alone and the direct burden of diseases due to acute COVID-19, but also to the sequelae and complications that the disease can leave among survivors [9,10]. The term long COVID-19 was first used by Perego in social media to denote the persistence of symptoms weeks or months after the initial SARS-CoV-2 infection, and the term ‘long haulers’ was used by Watson and by Yong [11].

It has been reported that most infectious diseases can cause post-acute, chronic, or long-term sequelae, lasting for weeks, months, or years, thus increasing the impact of this disease on individuals, communities, as well as on health systems around the globe [12,13,14]. The acute clinical manifestations of COVID-19 have been well characterized and involve both pulmonary and extrapulmonary systemic manifestations, but for some people, symptoms of COVID-19 persist beyond the acute setting [15]. These COVID-19-related sequelae can be attributed directly to the viral SARS-CoV-2 infection or the severe inflammatory response the body creates in response to the acute infection [9,16]. The World Health Organization defines post-COVID-19 or long COVID-19 as a condition that occurs in individuals with a history of probable or confirmed SARS-CoV-2 infection, generally three months after the appearance of COVID-19, with symptoms that last at least two months and cannot be explained by an alternative diagnosis [17].

In total, 87% of people recovered and discharged from hospitals showed persistence of at least one symptom even at 60 days [18]; the prevalence of residual symptoms was about 35% in patients treated for COVID-19 on an outpatient basis, but around 87% among cohorts of hospitalized patients [19]. The most common symptoms of long COVID-19 are extreme tiredness (fatigue), shortness of breath, loss of smell, and muscle aches [20,21]. However, there are plenty of symptoms one can have after a COVID-19 infection, including: problems with memory and concentration (brain fog), chest pain or tightness, difficulty sleeping (insomnia), heart palpitations, dizziness, pins and needles, joint pain, depression and anxiety, tinnitus, earaches, feeling sick, diarrhea, stomach aches, loss of appetite, high temperature, cough, headaches, sore throat, changes to sense of smell or taste, and rashes [11,21]. It is essential to point out that even though the severity of the disease is a significant risk factor, several patients with milder and asymptomatic processes can develop long-lasting and disabling sequelae, such as brain fog, paresthesia, muscle weakness, and others [22]. Despite advancing treatments for COVID-19, the long-term sequelae of this disease are expected to endure in survivors [23].

Studying, analyzing, and identifying the central sequelae related to COVID-19 are fundamental to understanding the real burden of COVID-19. Furthermore, we have to explore the economic and productive impact that long COVID-19 can generate, among the indirect consequences. This new condition will undoubtedly generate new doctor visits, higher out-of-pocket expenses, and more medication-related expenditures, affecting countrywide economies and patients’ quality-of-life [24,25].

The aim of this study was to present an overview of the information available on the sequelae of COVID-19 in people who have suffered from the infection.

## 2. Materials and Methods

### 2.1. Study Design

A systematic review was carried out in which observational descriptive, cohort studies, case series, and clinical case reports were included. The Prisma methodology was used, which is recommended for carrying out meta-analyses and systematic reviews [26,27,28].

### 2.2. Search Strategies

The search for information was carried out in both English and Spanish to obtain the maximum amount of information available. The Google search engine was used to access PubMed and Scielo databases, as well as the library system of the International University of Valencia to access SCOPUS and Web of Science databases. Information published from 2020 to 1 June 2022 was sought. The following terms were used to carry out said search:

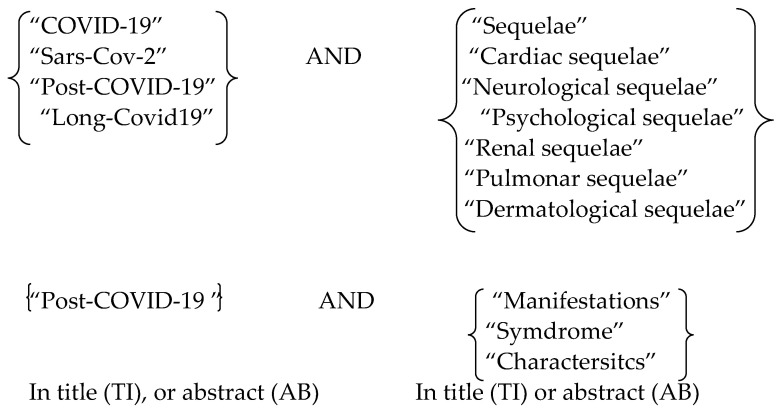



### 2.3. Inclusion Criteria

- Studies including COVID-19-recovered patients, either children or adults.
- Studies that meet the established follow-up time of long-term sequelae of COVID-19. The World Health Organization defines long COVID-19 as a condition that occurs in individuals with a history of probable or confirmed SARS-CoV-2 infection, generally three months after the appearance of COVID-19, with symptoms that last at least two months and cannot be explained by an alternative diagnosis [17].

### 2.4. Exclusion Criteria

- We excluded systematic, literature, and narrative reviews; letters to the editor; editorials; and meta-analyses. We also excluded those studies related to SARS or MERS.
- Animal studies and those investigations not reporting long COVID-19 sequelae were also excluded.

### 2.5. Data Synthesis

In this section, a complete and exhaustive review was carried out on all the manuscripts that met the inclusion criteria. With the information obtained from the cohort studies, a quantitative analysis was carried out, for which the Newcastle—Ottawa Quality Assessment Scale was used (Table S1). This scale was developed by the University of Newcastle and the University of Ottawa to assess the quality of studies by analyzing three aspects: selection of participants, comparability of study groups, and determination of the exposure or outcome of interest [29]. The studies evaluated with this scale had an acceptable to good quality, and to analyze the cross-sectional studies, the JBI Critical Appraisal Checklist for Analytical Cross-Sectional Studies was used (Table S2). In the case of the clinical cases (case reports), the JBI Critical Appraisal Checklist for Case Reports (Table S3) was used, and for case series, the JBI Critical Appraisal Checklist for Case Series was used (Table S4); this scale was developed by the Joanna Briggs Institute and allows evaluating the quality of a study, where a bias in its design, conduct, and analysis may have occurred [30,31,32]. Studies assessed with this scale were of moderate to high quality. At the end of this process, the information from the manuscripts was organized and synthesized in tables.

## 3. Literature Used

### 3.1. Literature Review

**Figure 1:**
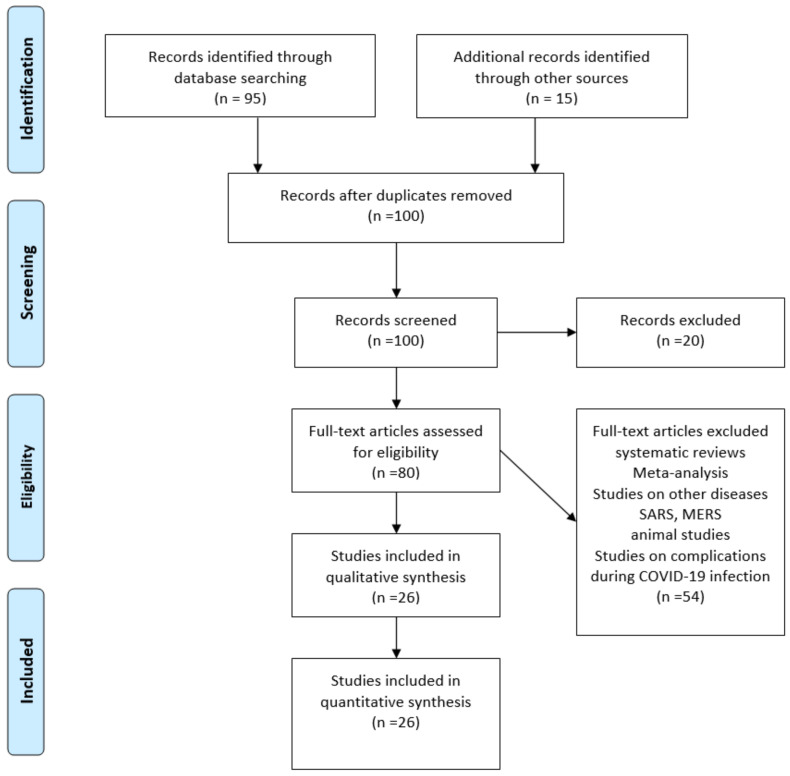
PRISMA flow chart.

During the first step of the literature review process, 100 manuscripts were included and appraised for compliance with the inclusion criteria. After this process, we obtained a remainder of 80 articles, in which we performed an analysis of the information provided within the abstracts. Finally, we selected 26 manuscripts for this literature review (Figure 1).

### 3.2. Specific Studies

#### 3.2.1. Pulmonary Sequelae

We included 8 studies for this section (Table 1). In these studies, patients had 3 to 12 months to have recovered from the COVID-19 infection. The study of the effects on lung function was assessed in 55 patients who had recovered from COVID-19 at least three months before the spirometry analysis [33,34]. The results demonstrated that 10.91% of patients had a decreased respiratory function as determined by a reduced forced expiratory volume in the first second (FEV1), and by a reduced forced vital capacity (FVC) [33,34]. In the same context, Huang et al., classified the degree of pulmonary dysfunction into 7 categories of a severity scale: 13% of those in category 5–6 had FEV1 < 80%, and 13% of those in category 4 had FEV1/FVC < 70% [35]. In the same study, a 59% reduction in carbon monoxide diffusion (Dlco) from its normal value was also reported. This anomaly correlated with the D-dimer value at hospital admission [36,37].

Pulmonary radiological alterations were also described by employing computed tomography scans (CT). Radiologic evidence demonstrated the presence of fibrosis affecting different lung sections, consolidating in certain lung lobes, mainly the right lower lobe, and the left upper and lower lobes [36,37]. Pan et al., conducted a study with 209 patients after one year of being diagnosed with COVID-19. The study reported the presence of multifocal reticular or cystic lesions in 13% of patients and evidence of residual linear opacities in 12% of them. Ground glass pattern, bronchial dilation, and parenchymal bands were the more common lesions during the acute and post-acute phases of the COVID-19 infection. The study also showed that 75% of patients did not show radiological alterations after one year [38].

It is essential to highlight the role of the severity of the infection. All of the available studies showed that the more severe the infection was, the greater the lung capacity alteration [35]. Although earlier mechanical ventilation had been associated with better survival among severely ill COVID-19 patients, it also behaved as a predictor of radiological and spirometric abnormalities [38,39].

Six studies highlighted the persistence of respiratory symptoms in patients 1 to 3 months after overcoming the COVID-19 infection. The most reported persistent symptom was dyspnea [12,36,39,40,41,42]. At least 65.6% of patients in the intensive care unit (ICU) and 42% of those hospitalized in general wards presented difficulty breathing after discharge [40]. Women and Afro-descendants reported higher dyspnea rates (42.1%) than white populations (25%). Lastly, patients with a body mass index (BMI) higher than 30 had more severe dyspnea than those with a BMI > 30 [40]. On top of dyspnea, other symptoms such as cough, runny nose, sneezing, rhinitis, burning sensation in the lungs and trachea, fatigue, and chest pain have been reported [36,39,41].

Comorbidities and the severity of COVID-19 were associated with a more significant respiratory impact. For example, having diabetes mellitus or hypertension increased the risk of developing pulmonary sequelae [12,35,38,39,40]. People from 40 to 60 years of age were twice as likely to present sequelae compared to younger patients [38,39].

**Table 1:** Summary of the literature mentioning pulmonary sequelae.

Author	Aims	Type of Study	Participants	Results	Score
Huang C. et al., 2021	To screen for long-term sequelae in patients discharged from hospital after COVID-19 and to identify potential risk factors, including disease severity, associated with these consequences [35].	Cohort study	1733 participants	They classified the severity of the disease they had into 7 categories through the severity scale, 13% of those in category 5–6 presented FEV1 < 80% and 13% of patients in category 4 presented FEV1/FVC < 70%.	8
			897 men		
			836 women		
			Age 47–65 years		
			6-month follow-up after hospital discharge.		
Halpin S. et al., 2020	To describe post-discharge symptoms and rehabilitation needs in COVID-19 survivors after hospital discharge [40].	Cross-sectional study	100 participants	- 72% of patients in the ICU and 60.3% hospitalized presented fatigue. - 65.6% of patients in the ICU and 42.6% hospitalized had dyspnea.	5
			32 patients in ICU		
			68 patients in hospital		
			33 women		
			35 men		
			Age 20–93 years		
			Follow-up 4 to 6 weeks after hospital discharge.		
Zhao Y. et al., 2020	To describe the long-term effects on changes in both lung function and CT images in patients with COVID-19 3 months after hospital discharge [36].	Cohort study	55 Participants	16.36% of the patients presented exertional dyspnea and 1.81% cough.	8
			23 women		
			32 men		
			Mean age 47.74 ± 15.49		
			Follow-up was 3 months after hospital discharge.		
Compagnone N. et al., 2019	To assess the pulmonary status of COVID-19 survivors after ICU discharge [37].	Cohort study	49 participants who were admitted to the ICU	- 46% of the patients presented a restrictive pattern after spirometry. - 28% presented radiological characteristics of pulmonary fibrosis.	7
			5 women		
			44 men		
			Age 52–65		
			Follow-up at 4, 12, and 24 weeks after hospital discharge.		
Pan F. et al., 2021	To assess chest CT manifestations of COVID-19 up to 1 year after symptom onset [38].	Cohort study	209 participants	- 53 patients presented alteration in pulmonary CT. - 77% of patients presented reticular lesions. - 74% of the patients presented bronchial dilation.	8
			116 women		
			93 men		
			Age 20–82 years		
			1-year follow-up after hospital discharge.		
Goërtz Y. et al., 2020	To assess whether multiple relevant symptoms recover after symptom onset in hospitalized and non-hospitalized patients with COVID-19 [41].	Cohort study	2113 participants	71% reported dyspnea and 24% pain in the lungs.	8
			102 hospitalized		
			2001 not hospitalized		
			1803 women		
			310 men		
			Age 39–54 years		
			Follow-up 3 months after recovery from COVID-19.		
Walsh M. et al., 2020	To investigate the prevalence and characteristics of prolonged symptoms in non-hospitalized college students experiencing mild to moderate acute illness [42].	Cohort study	148 male and female students	43% presented dyspnea.	7
			Middle age: 19.86 ± 3.03		
			Follow-up ≥ 28 days.		
Kamal M. et al., 2020	To investigate and characterize the manifestations that appear after the eradication of coronavirus infection and their relationship with the severity of the disease [12].	Cohort study	287 participants	28% presented dyspnea and 4.9% pulmonary fibrosis.	7
			184 women		
			103 men		
			Age 20 to 60 years		
			Follow-up does not specify how many days after having overcome the COVID-19 infection.		

#### 3.2.2. Cardiac Sequelae

In this section, we included 3 studies (Table 2). The cohort of long-term cardiological sequelae was evaluated in recovered patients after one to six months post-COVID-19 infection. Puntman et al., found that after 71 days post-SARS-CoV-2 infection, 78% of the patients had some evidence of cardiac compromise. For example, they had a lower ejection fraction in both the left and right ventricles; as well as higher pre-cardiac stroke volume; and higher levels of anti-troponin T1, T2, and troponin T [43]. Likewise, Huang et al., performed magnetic resonance (MRI) imaging on 36 patients who recovered from COVID-19 infection. They found significant alterations around native values of T1 and T2 with a significant reduction in the left ventricle’s ejection fraction [44]. These studies also reported the presence of focal myocardial fibrosis and the presence of active lymphocytic inflammation without evidence of another viral genome [43,44].

The most common cardiological symptoms after recovering from COVID-19 were palpitations [33,35,43,44,45]. Dyspneic patients, women, young adults, and those with heart rates ≥ 90 beats per minute were at higher risk of prolonging palpitations at resting [33]. In addition, chest pain, tightness, and thoracic distress were reported [13,33,35,44,45]. Newly diagnosed hypertension was reported among seven patients after 97 days post-infection, and the stroke risk increased among long haulers [13,33,46]. Sundari et at. used a technique that was aided with an SVM-based classifier for decision support via risk factor validation. The technique has provided an improved predictive accuracy and reliability over the risk factor validation caused due to pandemic parameters [47].

#### 3.2.3. Renal Sequelae

In this section, we included two studies (Table 3); Bowe et al., 2022, showed that after 30 days after recovery from COVID-19, patients had a significant reduction in the glomerular filtration rate of more than 30%, an estimated glomerular filtration reduction of ≤ 50%, and a higher risk of suffering from any urogenital and renal disease in the following months [48]. Huang et al., analyzed 1733 patients who recovered from COVID-19 infection. Their results showed that 35% had an estimated glomerular filtration rate of 90 mL/min per 1.73 m^2^: a lower rate than expected [35]. Although the lengthy hospitalizations and comorbidities may cause adverse effects on the kidneys, we cannot rule out that SARS-CoV-2 has some direct deleterious effect on the kidney [48].

#### 3.2.4. Neurological and Psychiatric

We included 8 studies in which participants were followed-up for up to 9 months post-infection (Table 4).

**Figure 2:**
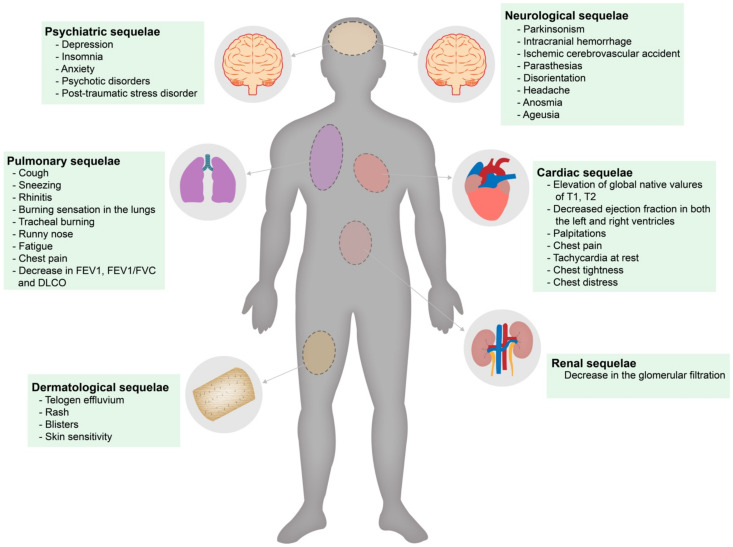
The main reported post-COVID-19 sequelae and long-term symptoms attributed to long COVID-19.

COVID-19 infection can cause neurological and psychiatric sequelae, including depression, insomnia, anxiety, and other disorders such as dementia and post-traumatic stress disorder [49,50,51,52,53]. In addition, Mazza et al., observed that lengthy hospital stays, the presence of comorbidities, and being women had higher scores for mental disorders [49]. Ahmed et al., used the detection and validation of voice signals through the identification of depression and mental disorder based on the processing of voice signals using a neural network; this technique was used to validate 2173 global voice samples, and the resulting accuracy of mental state and feelings was identified with 93.5% accuracy in classifying the behavioral patterns of patients suffering from COVID-19 and pandemic-influenced depression [54]. Among the least frequent neurological sequelae, Parkinsonism was reported on some occasions. An MRI performed on a patient with this sequela revealed that the periventricular white matter presented ischemic changes [50,55]. Additional long-term sequelae, including frequent headaches, paresthesias, disorientation, anosmia, and ageusia (Figure 2), were reported; furthermore, more severe complications such as intracranial hemorrhages and ischemic cerebrovascular events have been reported [50,52,56]. Mattioli et al., observed that recovered severely ill patients had significantly reduced tendon reflexes, areflexia, essential tremors, and affected tactile sensation [53]. In the same context, Mohmood et al., reported a case of a man whose physical examination revealed 2/5 left ankle strength at dorsiflexion and absent left and right knee reflexes. They also reported altered nerve conduction, active denervation, and axonal mononeuropathies [14]. Finally, Needham et al., reported a case series of 11 patients suffering from severe COVID-19, diagnosed with focal axonal loss and multiple mononeuritis [57].

**Table 4:** Summary of literature mentioning neurological and psychiatric effects.

Author	Aims	Type of Study	Participants	Results	Score
Rajaram R. et al., 2022	To present three cases of patients with COVID-19 who developed Parkinsonism and responded to levodopa [55].	Case report	3 patients	Parkinsonism.	6
			case 1: 72-year-old male without comorbidities.		
			case 2: 66-year-old male with diagnoses of high blood pressure, diabetes mellitus, and seizure disorder.		
			case 3: male case of 74 years.		
Mahmood S. et al., 2022	To describe a rare presentation of bilateral lower limb axonal mononeuropathies 2 weeks after recovery from COVID-19 pneumonia [14].	Case report	1 61-year-old male patient with a history of diabetes mellitus and arterial hypertension.	Axonal mononeuropathies of the lower limbs.	8
Taquet M. et al., 2021	To provide robust estimates of the incidence rates and relative risks of neurological and psychiatric diagnoses in patients within 6 months of a COVID-19 diagnosis [50].	Cohort study	236,379 participants	2.44% intracranial hemorrhage.	9
			131,460 women		
			104,015 men	1.97% ischemic stroke.	
			Average age of 49 years	1.42% Parkinsonism.	
			6-month follow-up after recovery from COVID-19 infection.	2.33% dementia.	
				1.48% insomnia.	
Furlan D. et al., 2020	To investigate the occurrence of psychiatric and cognitive impairments in a cohort of survivors of moderate or severe forms of COVID-19 [51].	Cohort study	425 participants		7
			205 women	8% depression.	
			220 men	15.5% anxiety.	
			Average age of 55 years	13.6% post-traumatic stress disorder.	
			Follow-up 6 to 9 months after recovery from COVID-19 infection.		
Romero D. et al., 2021	To identify and quantify the frequency and outcomes associated with the presence of sequelae or persistent symptoms during the 6 months after discharge for COVID-19 [52].	Cohort study	797 participants 369 women 428 men Average age 63 years Follow-up 6 months after hospital discharge.	6.8% anxiety. 4.9% sleep disturbance. 4.4% depression. 7.2% anosmia. 3.4% paresthesia.	7
Mattioli F. et al., 2022	To compare neurological and cognitive functions at four months follow-up in patients with mild to moderate COVID-19 versus patients requiring ICU admission [53].	Cohort study	215 participants	Neurological deficits in 1.2% of patients not admitted to the ICU and 13.5% of those admitted to the ICU.	7
			135 women		
			80 men		
			52 in ICU		
			163 not admitted to the ICU		
			Mean ICU age 60 years		
			Mean non-ICU age 46.9 years		
			Follow-up at 4 months after COVID-19 infection.		
Mattioli F. et al., 2021	To investigate the presence of focal neurological deficits as well as cognitive impairment in a group of patients with COVID-19, who were examined 4 months after diagnosis [56].	Cohort study	120 participants 90 women 20 men Age 26–65 years Follow-up 4 months after COVID-19 infection.	6.6% insomnia. 19.1% anosmia. 10.8% ageusia. 1.6% tremor.	9
Needham E. et al., 2020	The presentation of 11 cases of patients with COVID-19 who developed mononeuritis multiplex after being discharged from the ICU [57].	Case series	11 patients	Mononeuritis multiplex.	10
			8 men		
			11 women		
			Age 21–53 years.		

In terms of ethnicity, little is known about the epidemiological differences among populations. For instance, Asian patients were more likely to suffer from intracranial hemorrhage problems, while African Americans were more affected by Parkinsonism, and white participants had more cases of dementia and insomnia. In addition, the elderly (>65 years) had a higher risk of developing neurological and psychiatric sequelae [37]. As for the other organs and systems, the most severe COVID-19 comorbidities and infectious conditions were positively associated with neurological sequelae [52]. In contrast, sex differences were evidenced by women developing more headaches, paresthesias, anosmia, disorientation, confusion, and sleep disorders, while men reported more frequent paresthesias, anosmia, disorientation, confusion, and sleep disorders [52].

#### 3.2.5. Dermatological Sequelae

We included six studies in the dermatological section (Table 5). The most common affection reported was telogen effluvium, often reported between 1 to 3 months post-infection [58,59,60,61,62,63,64]. Other clinical findings were low hair density and softer hair, which marked positive in the traction test, and empty follicles using trichoscopy and Trichogramma [58,59,60,61,62,63,64]. The least common hair-loss pattern was bitemporal-frontal in 5.12% of cases, followed by front-vertical in 7.69%, and occipital in 12.82%; the most common pattern was the bitemporal and diffuse affection in 30.76% and 43.58%, respectively [59].

As telogen effluvium was the most common dermo-capillary complaint, it also was more often described by women. For example, Aksoy et al., observed that after COVID-19 recovery, 42.3% of women and 6.2% of men reported telogen effluvium [60]. A study including 204 patients observed that telogen effluvium developed in 57 patients, from which 31.7% had a hospitalization history, and 24.3% were outpatients [60]. The physiopathological mechanism behind hair loss is still unclear; however, a proinflammatory cascade with high levels of cytokines was postulated as the main reason, followed by the use of a multidrug regimen during the acute phase of COVID-19 [62,65]. Anya. J et al., reported a case series of 100 patients who recovered from COVID-19, 143 to 258 days ago. The results revealed that 26% had a rash, 8% had blistering, 15% had high skin sensitivity, and 13% reported alopecia [66].

**Table 5:** Summary of literature mentioning skin sequelae.

Author	Aims	Type of Study	Participants	Results	Score
Arenas C. and Diaz M., 2021	To present 2 patients who presented telogen effluvium as a manifestation of post-COVID-19 syndrome [58].	Case report	2 cases	telogen effluvium.	8
			First: 56-year-old female case with no pathological history.		
			Second case: a 16-year-old female with no pathological history.		
Sharquie K. and Jabbar R., 2021	To study the possible effects of COVID-19 on the hair growth cycle, and the relationship between COVID-19 and acute telogen effluvium [59].	Cross-sectional study	39 patients	telogen effluvium.	6
			36 women		
			3 men		
			Age 22–67 years		
			Follow-up 2 to 3 months after COVID-19 infection.		
Anaya J. et al., 2021	To report a series of patients with post-COVID-19 syndrome who attend a post-COVID-19 unit, and offer a comprehensive review on the subject [66].	Case series and comprehensive review	100 patients 53 women 47 men Age 37–58 years Follow-up at 143–258 days after COVID-19 infection.	26% rash. 8% ampoules. 15% skin sensitivity. 13% alopecia.	10
Aksoy H. et al., 2021	To assess the incidence of telogen effluvium developed after COVID-19 and the correlation between the development of telogen effluvium and the severity of the infection [60].	Cohort study	204 patients	27.9% telogen effluvium.	8
			123 women		
			81 men		
			Mean age 47.23 ± 16.471 years		
			3-month follow-up of positive PCR for COVID-19.		
Rizzetto G. et al., 2020	To report three cases of telogen effluvium occurring after a severe infection by SARS-CoV-2 [61].	Case report	3 patients	telogen effluvium.	8
			First female patient 62 years old, 3 months after recovery from infection.		
			Second female case, 74 years old, 3 months after hospital discharge.		
			Third female case, 58 years old, 3 months after hospital discharge.		
Rossi A. et al., 2021	To evaluate hair loss that occurs after SARS-CoV-2 infection using trichoscopy and trichogram to investigate possible patterns related to COVID-19 [63].	Case series	14 patients	telogen effluvium.	10
			12 women		
			3 men		
			Age 23–64 years		
			Follow-up from 1 to 3 months after COVID-19 infection.		

## 4. Discussion

The arrival of the SARS-CoV-2 virus caused illness and death worldwide. This topic of a global scope is relevant not only because of the acute burden of the disease, but also because of the long-term sequelae that have been reported [35,44,50,52]. The symptoms of prolonged COVID, like acute COVID-19, consist of a set of effects on various organs and systems such as the respiratory system. At the molecular level, the renin-angiotensin system is highly involved in the pathogenesis of this disease, and the expression of ACE2 receptors in various organs explains the diversity of prolonged symptoms of COVID-19. Other pathogenic mechanisms implicated in post-COVID-19 syndrome include immune-mediated vascular dysfunction, thromboembolism, and nervous system dysfunction [67,68].

We still do not know which factors are associated with the appearance of long COVID-19 or post-acute sequelae; however, it is known that more severe infections, as well as younger age, could be conditions that favor its appearance [69]. COVID-19 has caused a significant burden on the overstretched health systems. COVID-19 comes with dramatic and severe sequelae (cardio-respiratory, neurological, and psychiatric conditions), urging us to prioritize its attention globally. The fact that the disease is more common in young and young adult populations, and is primarily reported by women, suggests that the impact of the disease will be more significant than expected. Considering the expense related to medical care and healthcare services, the low productivity of individuals due to chronic fatigue, the over-the-counter medication abuse, and unnecessary out-of-pocket spending, the importance of long COVID-19 within our societies is predominant [12,13,14].

According to the results of Izquierdo et al., in their study, in which they analyzed long COVID-19 in Ecuador, patients with complete vaccination schedules and a booster constituted the majority of the long COVID-19 reports 70.6% (n = 5472); however, the highest proportion of symptoms was observed in the group that received only 1 dose compared to the rest of the groups (*p* > 0.05) [70].

The greater the number of studies, the greater our ability to counteract and prevent further impact. Although comorbidities have been linked to the natural history of COVID-19, very few studies have addressed this aspect. In a worldwide population, hypertension, obesity, diabetes, and other chronic illnesses such as asthma are becoming very common, as well as COVID-19, which does not seem to be slowing down in its transmission. The pandemic is not near an end, and younger populations are getting more and more infected. If we do not act rapidly, long COVID-19 can take a toll more significantly than expected [12,13,14]. In this same context, another prevalent factor to consider is overweight and obesity. In these already affected and service-consuming populations, long COVID-19 is more frequent [40]. Ethnicity must also be taken into account; Tachet et al., observed that Asian patients were more likely to suffer from intracranial hemorrhage problems, while African Americans were more affected by Parkinsonism, and white participants had more cases of dementia and insomnia [50].

In addition, longer follow-up studies are suggested. This is supported by Pan et al., who observed that patients 1 year after overcoming the acute infection still presented alterations in lung imaging studies, opening the possibility of several sequelae of COVID-19 with a long duration (greater than 12 months) [38].

The evaluation of the quality of the studies showed that the manuscripts that evaluated the development of dermatological sequelae had weaknesses, since of the 8 studies analyzed in this work, 5 corresponded to study designs with a limited utility such as clinical cases and case series [58,61,63,64,66]. On the other hand, regarding the study of renal sequelae, we consider that the available information was limited, since only 2 studies evaluated this aspect [35,48]. When comparing the strengths and weaknesses of these studies, it can be seen that the study conducted by Bowe et al., had 89,216 patients recovered from COVID-19 infection, considered a sufficient sample, but only carried out a 30-day follow-up period; while in the study by Huang et al., with a more limited sample of 1733 patients, a follow-up of 6 months after the acute infection was carried out. Details that reveal the wide differences and limitations of the studies on the sequelae of COVID-19 infection are currently available [35].

This study had several limitations, among which was that some of the symptoms that have been evaluated so far are subjective, such as chest pain or the sensation of palpitations. In addition, when analyzing the studies, care must be taken due to the heterogeneity between the populations studied, marked mainly by important differences in the follow-up period. In the studies included in this investigation, no studies were found that have reported the variants of the SARS-CoV-2 virus that affected the participants, limiting the possibility of associating the types of the variants of the virus with the risk of suffering sequelae. Taking into account the loss of infection of the participants, it can be speculated that they would be variants such as the Alpha, but the new variants that have emerged during the final stages of 2021 and 2022 must be considered, such as the Delta, Omicron, or XE.

## 5. Conclusions

Studies have shown that the main long-term sequelae produced by COVID-19 were those of a pulmonary origin, especially fatigue and tiredness. In addition, the characteristics of the patients, such as being women, represented a higher incidence of sequelae, especially dermatological and psychiatric sequelae; likewise, several studies concluded that age, the presence of comorbidities, high BMI, and the severity of the acute COVID-19 infection predisposed a patient to the appearance of sequelae from COVID-19. However, studies with more robust methodologies and longer follow-up periods are needed to better understand the behavior of the sequelae of acute COVID-19 infection. To improve our understanding of long-term COVID-19 sequelae, it is recommended that future research be carried out globally in low and middle-income countries, and it is important to carry out research in the pediatric population and in pregnant women.

## Figures and Tables

**Table 2:** Summary of literature mentioning cardiac sequelae.

Author	Aims	Type of Study	Participants	Results	Score
Huang C. et al., 2021	To describe the long-term consequences of COVID-19 in patients after hospital discharge and identify potential risk factors, including disease severity, associated with these consequences [35].	Cohort study	1733 participants, 897 men, 836 women, age 47–65 years.	9% presented palpitations and 5% chest pain.	8
			6-month follow-up after hospital discharge.		
Puntmann V. et al., 2020	To evaluate the presence of myocardial damage in unselected patients recently recovered from COVID-19 disease [43].	Cohort study	100 participants, 47 women, 53 men. Average age 49 years, follow-up from 64 to 92 days after recovery from COVID-19 infection.	60% myocardial inflammation. 17 patients reported chest pain. 20 reported palpitations.	7
Xiong Q. et al., 2021	To describe the prevalence, nature, and risk factors of the main clinical sequelae in survivors of COVID-19 with more than 3 months of hospital discharge [38].	Longitudinal study	538 participants, 293 women, 245 men. Age 41–62 years.	12.3% presented pain in the chest, and 11.2% tachycardia at rest.	7
			The time from hospital discharge was 95 to 102 days.		

**Table 3:** Summary of literature mentioning renal sequelae.

Author	Aims	Type of Study	Participants	Results	Score
Bowe B. et al., 2021	To characterize the risks of post-acute renal outcomes according to the severity of acute COVID-19 infection [48].	Cohort study	89,216 participants	Acute kidney injury, and decreased estimated glomerular filtration rate.	9
			8817 women		
			80,399 men		
			Age 53–73 years		
			Follow-up 127–268 days after COVID-19 infection.		
Huang C. et al., 2021	To describe the long-term consequences of COVID-19 in patients after hospital discharge, and identify potential risk factors, including disease severity, associated with these consequences [35].	Cohort study	1733 participants	35% presented decreased glomerular filtration rate.	8
			897 men		
			836 women		
			Age 47–65 years		
			6-month follow-up after hospital discharge.		

## Data Availability

Not applicable.

## Supplementary Materials

The following supporting information can be downloaded at: https://www.mdpi.com/article/10.3390/healthcare10122364/s1, Table S1: Newcastle—Ottawa Quality Assessment Scale. Table S2: JBI Critical Appraisal Checklist for Case Reports. Table S3: JBI Critical Appraisal Checklist for Case Series. Table S4: JBI Critical Appraisal Checklist for Analytical Cross-Sectional Studies.
