# Supplementary material for: A Systematic Review and Quality Evaluation of Studies on Long-Term Sequelae of COVID-19
Source: Healthcare (Basel). 2022 Nov 24;10(12):2364. doi: 10.3390/healthcare10122364 (PMC9778202; doi:10.3390/healthcare10122364)
Supplement: Supplementary file 1 [file healthcare-10-02364-s001.zip › healthcare-1994474-supplementary.pdf]

healthcare

MDPI

Table S1. NEWCASTLE - OTTAWA QUALITY ASSESSMENT SCALE.

Study	1	2	3	4	5	6	7	8	Score
**Huang. Ch et al. 2021 [1]**	*		*	*	**	*	*	*	8
**Zhao. Y et al.2020 [2]**	*		*	*	**	*	*	*	8
**Compagnone. N et al. 2019 [3]**	*		*	*	*	*	*	*	7
**Pan. F et al. 2021 [4]**	*		*	*	**	*	*	*	8
**Goërtz. Y et al. 2020 [5]**	*		*	*	**	*	*	*	8
**Walsh. M et al. 2020 [6]**	*		*	*	*	*	*	*	7
**Kamal. M et al. 2020 [7]**	*		*	*	*	*	*	*	7
**Puntmann. V et al. 2020 [8]**	*		*	*	*	*	*	*	7
**Xiong. Q et al. 2021 [9]**	*		*	*	*	*	*	*	7
**Bowe. B et al. 2021 [10]**	*	*	*	*	**	*	*	*	9
**Taquet. M et al. 2021 [11]**	*	*	*	*	**	*	*	*	9
**Romero. D et al. 2021 [12]**	*		*	*	*	*	*	*	7
**Mattioli. F et al. 2021 [13]**	*	*	*	*	**	*	*	*	9
**Furlan. D et al. 2020 [14]**	*		*	*	*	*	*	*	7
**Mattioli. F et al. 2022 [15]**	*		*	*	*	*	*	*	7
**Aksoy. H et al. 2021 [16]**	*		*	*	**	*	*	*	8

Note: A study can receive a maximum of one star for each item numbered within the Selection and Result categories. A maximum of two stars can be awarded for comparability.

Selection

1. Representativeness of the exposed court.
2. Selection of the unexposed court.
3. Exposure determination.
4. Demonstration that the current outcome of interest was not present at baseline.

Comparability

5. Cohort comparability based on design or analysis.

Results

6. Evaluation of the result.
7. Was the follow-up long enough for the results to occur?
8. Adequacy of cohort follow-up.

Interpretation

Good quality: 3 or 4 stars in the selection domain and 1 or 2 stars in the comparability domain and 2 or 3 stars in the outcome/exposure domain.

Acceptable quality: 2 stars in the selection domain and 1 or 2 stars in the comparability domain and 2 or 3 stars in the outcome/exposure domain.

Poor quality: 0 or 1 star in the selection domain or 0 stars in the comparability domain or 0 or 1 stars In the outcome/exposure domain

Table S2: JBI Critical Appraisal Checklist for Case Reports.

Study	1	2	3	4	5	6	7	8	Score
**Rajaram. R et al. 2022 [17]**	YES	YES	YES	YES	YES	NO	NO	YES	6
**Mahmood. S et al. 2022 [18]**	YES	YES	YES	YES	YES	YES	YES	YES	8
**Arenas. C y Diaz. M 2021 [19]**	YES	YES	YES	YES	YES	YES	YES	YES	8
**Rizzetto. G et al. 2020 [20]**	YES	YES	YES	YES	YES	YES	YES	YES	8
**Otsuka. Y et al. 2022 [21]**	YES	YES	YES	YES	YES	YES	YES	YES	8

1. Were patient demographics clearly described?
2. Was the patient's history clearly described and presented as a timeline?
3. Was the patient's current clinical condition clearly described at the time of presentation?
4. Were diagnostic tests or evaluation methods and results clearly described?
5. Were interventions or treatment procedures clearly described?
6. Was the post-intervention clinical condition clearly described?
7. Were adverse (harm) or unforeseen events identified and described?
8. Does the case report provide lessons to take away?

Table S3: JBI Critical Appraisal Checklist for Case Series.

Study	1	2	3	4	5	6	7	8	9	10	Score
**Needham. E et al. 2020 [22]**	YES	YES	YES	YES	YES	YES	YES	YES	YES	YES	10
**Anaya. J et al. 2021 [23]**	YES	YES	YES	YES	YES	YES	YES	YES	YES	YES	10
**Rossi. A et al. 2021 [24]**	YES	YES	YES	YES	YES	YES	YES	YES	YES	YES	10

1. Were there clear criteria for inclusion in the case series?
2. Was the condition measured in a standard and reliable way for all participants included in the case series?
3. Were valid methods of condition identification used for all participants included in the case series?
4. Did the case series include consecutive participants?
5. Did the case series have complete inclusion of participants?
6. Were there clear reports on the demographics of the study participants?
7. Was there a clear reporting of the clinical information of the participants?
8. Were outcomes or case follow-up results clearly reported?
9. Was there clear reporting of demographic information from the presenting sites/clinics?
10. Was the statistical analysis appropriate?

Table S4: JBI critical appraisal checklist for analytical cross-sectional studies.

Study	1	2	3	4	5	6	7	8	Score
**Halpin. S et al. 2020 [25]**	YES	YES	YES	NO	NO	NO	YES	YES	5
**Sharquie. K y Jabbar. R 2021 [26]**	YES	YES	YES	YES	NO	NO	YES	YES	6

1. Were the criteria for inclusion in the sample clearly defined?
2. Were the study subjects and setting described in detail?
3. Was the exposure measured in a valid and reliable way?
4. Were standard and objective criteria used to measure the condition?
5. Were confounding factors identified?
6. Were strategies established to deal with confounding factors?
7. Were the outcomes measured in a valid and reliable way?
8. Was an appropriate statistical analysis used?
